# Bioelectrochemical conversion of CO_2_ to value added product formate using engineered *Methylobacterium extorquens*

**DOI:** 10.1038/s41598-018-23924-z

**Published:** 2018-05-08

**Authors:** Jungho Jang, Byoung Wook Jeon, Yong Hwan Kim

**Affiliations:** School of Energy and Chemical Engineering, Ulsan National Institute of Science and Technology (UNIST), 50 UNIST-gil, Ulsan, 44919 Republic of Korea

## Abstract

The conversion of carbon dioxide to formate is a fundamental step for building C1 chemical platforms. *Methylobacterium extorquens* AM1 was reported to show remarkable activity converting carbon dioxide into formate. Formate dehydrogenase 1 from *M. extorquens* AM1 (MeFDH1) was verified as the key responsible enzyme for the conversion of carbon dioxide to formate in this study. Using a 2% methanol concentration for induction, microbial harboring the recombinant MeFDH1 expressing plasmid produced the highest concentration of formate (26.6 mM within 21 hours) in electrochemical reactor. 60 μM of sodium tungstate in the culture medium was optimal for the expression of recombinant MeFDH1 and production of formate (25.7 mM within 21 hours). The recombinant MeFDH1 expressing cells showed maximum formate productivity of 2.53 mM/g-wet cell/hr, which was 2.5 times greater than that of wild type. Thus, *M. extorquens* AM1 was successfully engineered by expressing MeFDH1 as recombinant enzyme to elevate the production of formate from CO_2_ after elucidating key responsible enzyme for the conversion of CO_2_ to formate.

## Introduction

The rapid development of a wide-range of industrial technologies using fossil fuel has led to drastically increased level of atmospheric carbon dioxide (CO_2_)^1^. Furthermore, a vast number of reports confirm that elevated carbon dioxide level in the atmosphere can be a determining factor of global warming via greenhouse effects^1–3^. The conversion of CO_2_ to value-added chemicals has been posited as an indispensable technology to slow down the rate of atmospheric [CO_2_] accumulation^4,5^. Fortunately, CO_2_ is a promising renewable source to produce environmentally friendly chemical platforms such as formate, dimethyl carbonate, its polymers and others^6^.

Among many candidates that could be produced from CO_2_ as a feedstock, formate is reported as one of the most desirable bulk chemicals in terms of economic and environmental benefits^7^. Especially, direct formate fuel cell (DFFC) converting formate to electrical energy is a solution for storing renewable energy because formate can easily store energy generated from various renewable sources such as wind, solar, and hydro. Additionally, formate can be safely transported compared with hydrogen gas because formate is non-flammable, non-toxic and moderately inert in environment^8,9^.

Despite promising potential, conventional conversion technologies for CO_2_ into valuable compounds harbor critical limitations such as harsh reaction conditions and requirements of rare precious metal catalysts and expensive reducing agents such as hydride and hydrogen^10,11^. Even though electro-catalysis of carbon dioxide is a promising alternative given relative low costs of electrical energy, some chemical electro-catalysts frequently demonstrate insufficient reaction selectivity during the conversion of carbon dioxide to formate due to the production of significant ratio of hydrogen gas as by-product^12^. Accordingly, enzyme based electro-catalyzed production of formate from CO_2_ has received greater attention due to exceptional selectivity to formate^13–17^. However, effective oxygen tolerant biocatalysts capable of utilizing electrons supplied from cathode have been sought to render biocatalytic formate production from CO_2_ feasible^18^.

Previously, our group reported a new approach for the conversion of CO_2_ to formate through electro-catalysis using a whole-cell biocatalyst. *Methylobacterium extorquens* AM1 (*M. extorquens* AM1) as a whole-cell catalyst showed excellent formate production capability from CO_2_ with high oxygen-stability in electrochemical reactors. However, the enzyme primarily responsible for the synthesis of formate from CO_2_ remained unknown. Previous studies were focused on the optimization of reaction conditions such as electron mediators, cell mass concentration and pH value^19^.

In this study, we clearly demonstrate that formate dehydrogenase 1 (MeFDH1), a heterodimeric protein composed of alpha and beta subunits, is the essential enzyme in formate synthesis from CO_2_ in *M. extorquens* AM1 cells. The MeFDH1 coding gene-knockout verified the role of this enzyme in the synthesis of formate from CO_2_. In addition, we generated a homologous recombinant *M. extorquens* AM1 expressing gene of MeFDH1 tagged with hexahistidine. Recombinant *M. extorquens* AM1 not only provide higher MeFDH1 expression than wild-type, but also allow us to confirm optically the expression level of MeFDH1 through Western blotting. Furthermore, the His-tag of recombinant MeFDH1 enables the easy and high purity purification using Ni-NTA resin which is useful for further study on the characteristics of MeFDH1 in the future. Various factors including the concentration of metal ions (tungstate) and induction time using methanol as inducer were optimized for improved MeFDH1 expression and increased formate conversion from CO_2_ in MeFDH1 recombinant *M. extorquens* AM1.

## Results and Discussion

### Determination of essential enzymes for formate production from CO_2_ in *M. extorquens* AM1

**Figure 1:**
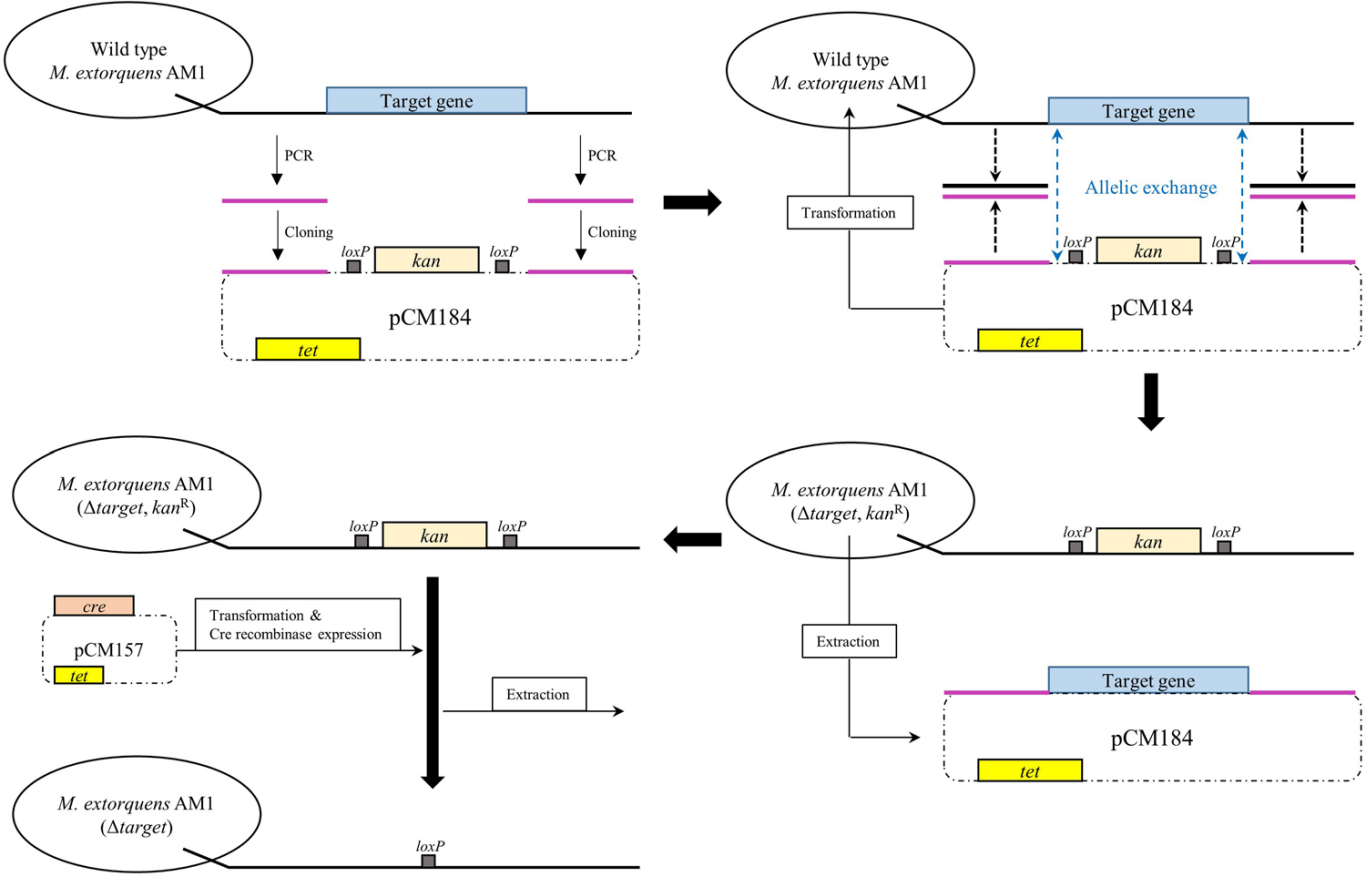
Diagram of the gene-knockout system for the construction of *M. extorquens* AM1 mutants. Sequences located on both sides of the target gene in the genome of *M. extorquens* AM1 were amplified by PCR and cloned into pCM184. The target gene was exchanged with the *loxP-kan-loxP* gene through homologous allelic recombination between *M. extorquens* AM1 and pCM184. For extraction of pCM184, *M. extorquens* AM1 mutants (Δtarget, KanR) were cultivated in media without kanamycin. Next, *cre* recombinase was expressed on pCM157 to eliminate the kanamycin resistance gene between *loxP* genes through site-specific recombination.

**Figure 2:**
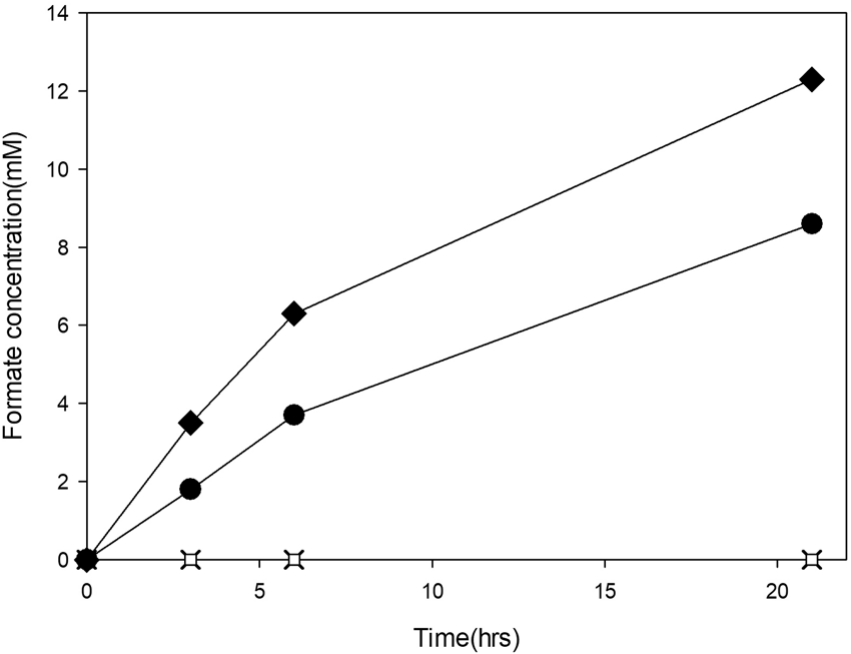
Formate production of strains in the electrochemical reaction system: F1A-P1 (⬥), WT (●), F1A (X), F1AB-P1B (□), reaction conditions: 0.6 g wet-cell, 10 mM MV, pH 6.0; CO_2_ gas purging (99.999%, rate: 1 mL/s).

The *M. extorquens* AM1 genome is known to contain four formate dehydrogenase coding genes (*fdh1*, *fdh2*, *fdh3* and *fdh4*)^20,21^. Among these formate dehydrogenase coding genes, the *fdh1* gene for MeFDH1 (GenBank accession No. ACS42636.1(α-subunit), ACS42635.1(β-subunit)) was selected for the generation of *M. extorquens* AM1 knock-out mutants because it was previously reported to be the principal formate dehydrogenase during whole-cell oxidation of supplied formate^21^. Accordingly, three species of *M. extorquens* AM1 mutants summarized in Table 1 were generated (Fig. 1). As shown in Fig. 2, while wild type (WT) and the MeFDH1 recombinant expression mutant (F1A-P1) could synthesize formate from CO_2_ in the electrochemical reaction system, neither the MeFDH1 alpha knockout mutant (F1A) nor the MeFDH1 beta knockout mutant (F1AB-P1B) produced any detectible level of formate from CO_2_. These results support the hypothesis that MeFDH1 is the key enzyme responsible for the conversion of CO_2_ to formate. In addition, both the α- and β-subunits of MeFDH1 were proven simultaneously required for MeFDH1 to function properly. The α-subunit of MeFDH1 (MeFDH1α) is reported to contain binding sites for bis-tungstopterin guanine dinucleotide cofactor, three 4Fe4S clusters and one 2Fe2S cluster. The β-subunit of MeFDH1 (MeFDH1β) harbors binding sites for flavin mononucleotide (FMN), NAD^+^ and one 4Fe4S cluster^22^. A purified recombinant β-subunit of MeFDH1 at atmosphere was successfully applied for the regeneration of NADH by utilizing methyl viologen as an artificial electron mediator in the electrochemical reactor^23^. This result implies that the β-subunit of MeFDH1 is essential for the reduction of CO_2_ catalyzed by MeFDH1 since FMN of MeFDH1β may be the sole site to accept electrons from MV.

**Table 1:** Bacterial strains and their plasmids for knockout or for recombinant expression.

Strain	Deletion gene	Knock-out plasmid	Recombinant plasmid	Selective antibiotic
Wild type	—	—	—	Rif
F1A	Δ*fdh1α*	pCM184(Δ*fdh1α*)	—	Rif, Kan
F1A-P1	Δ*fdh1α*	pCM184(Δ*fdh1α*) pCM157(*cre*)	pCM110(*fdh1*)	Rif, Tet
F1AB-P1B	Δ*fdh1αβ*	pCM184(Δ*fdh1α*), pCM157(*cre*), pCM184(Δ*fdh1β*)	pCM110(*fdh1α*)	Rif, Kan, Tet

The *M. extorquens* AM1 mutant (F1A-P1) showed higher formate production (0.98 mM/hr/g-wet cell) compared to the formate production (0.68 mM/hr/g-wet cell) in WT *M. extorquens* AM1, as shown in Fig. 2. The F1A-P1 strain harbored a homologously expressed recombinant MeFDH1 coded by pCM110(*fdh1*) since plasmid pCM110 contains *P*_*mxaF*_, which was reported as a strong inducible promoter in *M. extorquens* AM1. This promoter can significantly increase the expression of MeFDH1 because of its high promoter strength compared to other promoters^24^. Based on results of Fig. 2, we supposed that MeFDH1 expression *in vivo* can directly affect formate production.

### Confirmation of expressed recombinant MeFDH1 in *M. extorquens* AM1 (F1A-P1)

**Figure 3:**
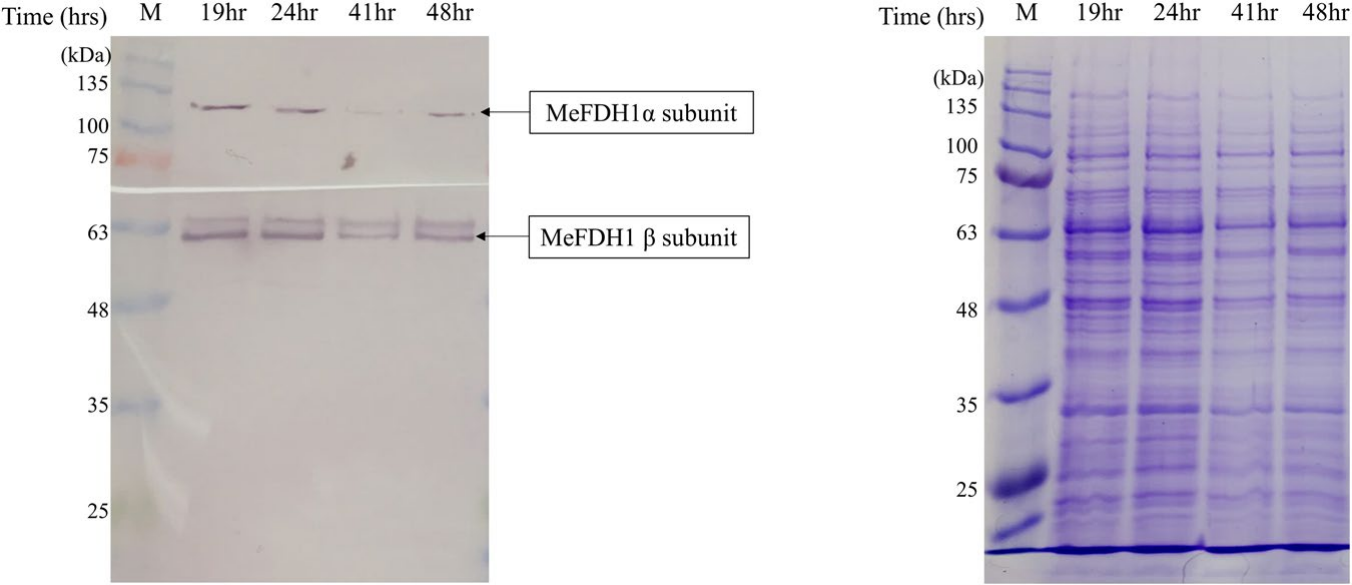
Time profile of MeFDH1 expression level. (**a**) SDS-PAGE (10%) of mutant (F1A-P1) crude extracts, (**b**) Western blotting of mutant (F1A-P1) crude extracts: protein marker (M), incubation times (19 hr, 24 hr, 41 hr, 48 hr).

The crude lysate of homologously expressed recombinant (F1A-P1) was used for SDS-PAGE and Western blotting to analyze the expression level of MeFDH1 in *M. extorquens* AM1 (F1A-P1). The target bands for MeFDH1α and MeFDH1β were difficult to distinguish due to the their relatively weak expression in SDS-PAGE (see Fig. 3(a)) despite molecular weight estimates of 108 kDa and 62 kDa, respectively. However, the α-subunit and β-subunit of MeFDH1 were clearly confirmed through western blotting as shown in Fig. 3(b). Interestingly, expression of MeFDH1α appeared slightly decreased after the 41 hours incubation, even though the recovered expression level was observed repeatedly at 48 hours. These observations imply that a considerable fraction of homologously expressed recombinant MeFDH1 can be degraded through endogenous metabolism^25^.

### Optimized culture conditions for increased MeFDH1 expression of mutant (F1A-P1) and formate production in an electrochemical CO_2_ reduction system

**Figure 4:**
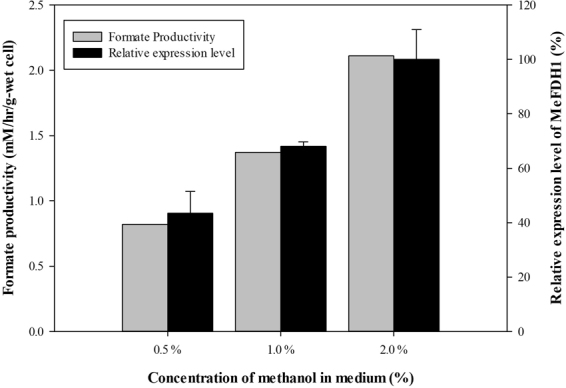
Relative expression level of MeFDH1 depending on methanol concentration and formate productivity in electrochemical CO_2_ reduction system (0.6 g wet-cell, 10 mM MV, pH 6.0; CO_2_ gas purging (99.999%, rate: 1 mL/s)).

Methanol was added to the culture medium to induce the expression of MeFDH1, as well as to supply a carbon source for *M. extorquens* AM1 since expression of MeFDH1 is controlled by the methanol-inducible promoter *P*_*mxaF*_^24^. The methanol concentration in the culture medium affected MeFDH1 expression in mutant (F1A-P1). Figure 4 shows that the higher methanol concentration produces greater MeFDH1 expression in mutant (F1A-P1), especially after a 48 hours incubation. As predicted, mutant (F1A-P1) cultured in medium with initial 2.0% methanol concentration showed 2.11 mM-formate/hr/g-wet cell as the highest formate production rate in the electrochemical CO_2_ reduction system. This result strongly supports our hypothesis that MeFDH1 expression has a proportional relationship with the formate production through CO_2_ reduction. In other words, much higher expression of MeFDH1 is expected to result in the higher production of formate since recombinant MeFDH1 is the critical limiting biocatalyst.

**Figure 5:**
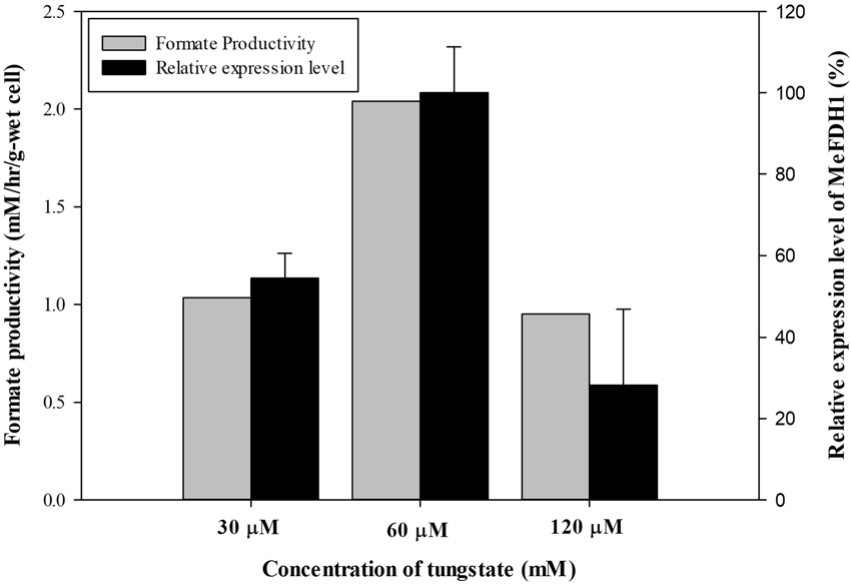
Relative expression level of MeFDH1 depending on cofactor (W) concentration and formate productivity in electrochemical CO_2_ reduction system (0.6 g wet-cell, 10 mM MV, pH 6.0; CO_2_ gas purging (99.999%, rate: 1 mL/s)).

Unlike other common formate dehydrogenases, MeFDH1 is known to contain W-pterin guanidine dinucleotide instead of Mo as its prosthetic group^21^. Generally, the availability of tungsten is reported to be much lower compared to its counterpart molybdenum^26^. Even though chemical properties of W and Mo are known to be quite similar, microbes appear to prefer one over the other based on its specific transporter^27^. Tungstate concentration initially added in the culture medium also affected the MeFDH1 expression level of mutant (F1A-P1). As shown in Fig. 5, mutant (F1A-P1) cultured in 2x tungstate (60 μM) resulted in increased expression of MeFDH1. However, 4x tungstate (120 μM) did not increase the expression of MeFDH1. This result may be related to observations that high concentrations of tungstate and W-pterin guanidine dinucleotide cofactor intermediates in the pterin pathway can cause cellular toxicity^28,29^. Interestingly, optimum concentration of tungstate seems to repress the degradation of recombinant MeFDH1 (Figure S1 of Supplementary Information), which implies that tungstate deficiency may cause improper production of apoprotein, and apoprotein may be more vulnerable to endogenous degradation^30^.

An artificial electron mediator was used for the electron transfer from copper plate cathode to MeFDH1 in electrochemical CO_2_ reduction system. Among many electron mediators, the mutant (F1A-P1) produced formate from CO_2_ when methyl viologen (MV) and ethyl viologen (EV) were employed as electron mediator, on the while other mediator such as FMN and NR did not work with the mutant (F1A-P1). (Figure S2 of Supplementary Information). Additionally, there are other papers reporting that MV was most suitable electron mediator for CO_2_ reduction reaction in electrochemical system using tungsten containing formate dehydrogenase^31,32^.

**Figure 6:**
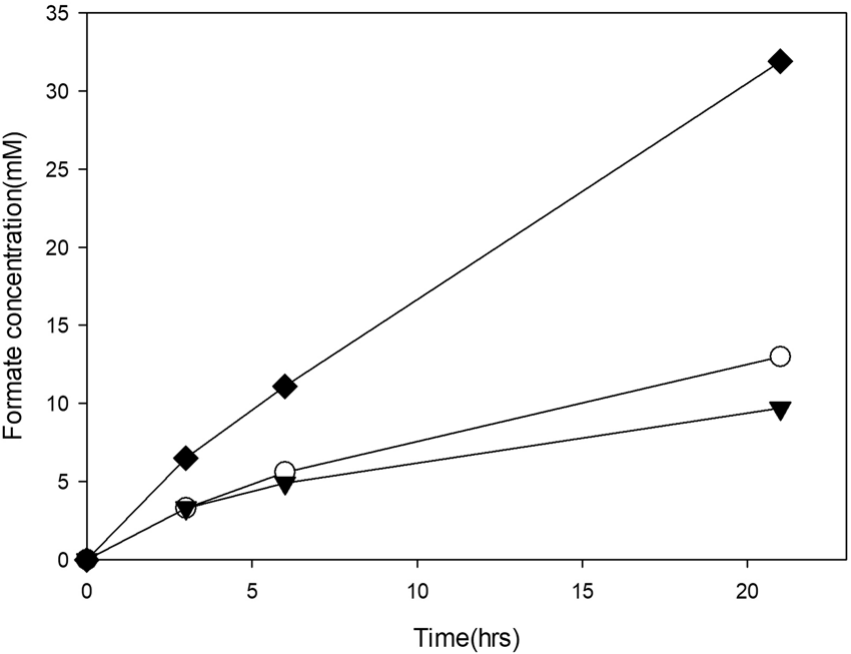
The comparison between formate production of wild type and that of mutant (F1A-P1) cultured under optimum conditions: WT cultured in basic conditions (0.5% (v/v) MeOH, 1x tungstate(30 μM)) (○), WT cultured in optimum conditions (2.0% (v/v) MeOH, 2x tungstate(60 μM)) (▼), F1A-P1 cultured in optimum conditions (2.0% (v/v) MeOH, 2x tungstate(60 μM)) (0.6 g wet-cell, 10 mM MV, pH 6.0; CO_2_ gas purging (99.999%, rate: 1 mL/s)) (⬥).

Based on the experimental results shown in Figs 4 and 5, the balance between the expression level of the apo-form and the flux rate of its cofactor pathway may be an indispensable requirement to obtain a significant quantity of the holo-form of MeFDH1 in *M. extorquens* AM1. The mutant (F1A-P1) produced over 30 mM of formate from carbon dioxide within 24 hours in electrochemical reactions. This was three times greater than the production of wild type *M. extorquens* AM1 at optimal methanol and tungstate concentrations (Fig. 6). Wild-type *M. extorquens* AM1, however, showed 0.77 mM/hr/g-wet cell, which is three times lower than that of recombinant mutant (F1A-P1). Furthermore, a less significant difference between optimum conditions and previous conditions was observed (Fig. 6). This result demonstrates that in contrast with the promoter *P*_*mxaF*_ of recombinant mutant (F1A-P1), the unknown promoter of the gene encoding MeFDH1 of *M. extorquens* AM1 wild type was not affected by methanol or tungstate concentrations in culture medium. Until now, the production of formate from carbon dioxide was proportional to the level of expression in recombinant MeFDH1. Consequently, a strong and simply regulated promoter such as *P*_*mxaF*_ was a core factor for homologous expression of recombinant MeFDH1 with an optimal tungstate concentration in the medium.

## Conclusion

The genetic engineering of *M. extorquens* AM1 and optimization of culture conditions allowed improved formate production in electro-biocatalysis. Importantly, this study demonstrated that MeFDH1 is a critical enzyme in the catalysis of carbon dioxide to produce formate. Furthermore, the expression level of recombinant MeFDH1 and the tungstate concentration as a MeFDH1 cofactor significantly improved microbial formate production. Engineered *M. extorquens* AM1 demonstrated enhanced production of formate, reaching 2.53 mM/hr/g-wet cell, 2.5 times greater than wild type *M. extorquens* AM1. These results clearly showed the microbe genetic engineering is able to enhance whole-cell biocatalyst activity.

## Methods

### Materials

*Methylobacterium extorquens* AM1 (ATCC 14781, GenBank accession No. CP001510.1) was used in this study. Culture medium was composed of a carbon source (16 g/L succinate) and minimal salt medium supplemented with trace elements and sodium tungstate. Minimal salt medium contained 1.62 g/L NH_4_Cl, 0.2 g/L MgSO_4_, 2.21 g/L K_2_HPO_4_, and 1.25 g/L NaH_2_PO_4_∙2H_2_O. Trace elements contained 15 mg/L Na_2_EDTA_2_∙H_2_O, 4.5 mg/L ZnSO_4_∙7H_2_O, 0.3 mg/L CoCl_2_∙6H_2_O, 1 mg/L MnCl_2_∙4H_2_O, 1 mg/L H_3_BO_3_, 2.5 mg/L CaCl_2_, 0.4 mg/L of Na_2_MoO_4_∙2H_2_O, 3 mg/L FeSO_4_∙7H_2_O, and 0.3 mg/L CuSO_4_∙5H_2_O. After incubation for 19 hours, methanol (final concentration: 124 mM) was added as an inducer. The final concentration of antibiotics was 50 μg/mL rifamycin (Rif), 50 μg/mL kanamycin (Kan) and 10 μg/mL tetracycline (Tet) in culture medium. Incubation proceeded at 26 °C in 1 L Erlenmeyer shake flasks (200 mL working volume) in a 200-rpm shaking incubator.

### Gene-knockout system and one-step sequence-and ligation-independent cloning (SLIC)

Gene-knockout was performed as described previously^33^. DNA located both upstream and downstream of tMeFDH1 (GenBank accession No. ACS42636.1(α-subunit), ACS42635.1(β-subunit)) was amplified by PCR. These sequences were cloned to locate both sides of the *loxP* and kanamycin genes of pCM184 (Addgene plasmid 46012). When *M. extorquens* AM1 was transformed with pCM184, allelic exchange occurred and *M. extorquens* AM1 acquired *loxP* and kanamycin genes but lost a partial gene sequence of MeFDH1. When *M. extorquens* AM1 was transformed with pCM157 (Addgene plasmid 45863), *cre* recombinase expressed on pCM157 extracted the kanamycin gene between the *loxP* sites through site-specific recombination. When *M. extorquens* AM1 was transformed with pCM110 containing MeFDH1 gene, MeFDH1 expression of *M. extorquens* AM1 was recovered. For all cloning, one-step SLIC was applied^34^. SLIC uses T4 DNA polymerase as exonuclease. This vector was linearized by restriction enzymes and sequences generated by PCR. NEB 2.1 buffer (B7202S, BioLabs) and T4 polymerase was then added. This mixture was incubated at room temperature for 2.5 min, then immediately transferred to ice for 10 min. After 10 min, 1 μl of mixture was added to 100 μl of competent E. coli DH5α cells (RBC). DH5α cells were incubated on ice for 20 min. Then, 950 μl of LB broth was added and incubated at 37 °C for 16 hours. *M. extorquens* AM1 mutants generated by gene-knockout are summarized in Table 1.

### Western blot analysis for MeFDH1 expression level

Cells were lysed with urea buffer (6 M urea, 200 mM NaCl, 20 mM Tris, pH 8.0) and crude extract was separated on SDS-PAGE (10% Tris/glycine) and transferred to PVDF membrane (Cat. No. KDM20, 10 cm × 10 cm, KOMA BIOTECH) through semi-dry transfer (AE-8130, ATTA) with transfer buffer (24.9 mM Tris, 2.5 M methanol, 191, 8 mM glycine, pH 8.4). After transfer, the membrane was wetted with blocking buffer (PBST; 10 mM phosphate buffer, 2.7 mM KCl, 137 mM NaCl, 1% (w/v) Tween 20) (2% (w/v) skim milk) and was gently shaken for 1 hour. The membrane was washed 4 times for 20 min in PBST buffer and then transferred to blocking buffer mixed with primary antibody and gently shaken for 1 hour. Next, the membrane was washed 4 times for 20 min in PBST buffer and then transferred to blocking buffer mixed with secondary antibody and gently agitated for 1 hour. Finally, the membrane was washed 4 times for 20 min in PBST buffer and then stained with BCIP®/NBT Liquid substrate solution (B1911, SIGMA). For the MeFDH1 alpha subunit, the primary antibody was Anti-6x His tag antibody (ab18184, ABCAM) (1:1000 dilution), and the secondary antibody was rabbit anti-mouse (ab6729, ABCAM) (1:2000 dilution). For the MeFDH1 beta subunit, a customized primary antibody was used (ABFRONTIER) (1:1000 dilution) and the secondary antibody was goat anti-rabbit (ab6722, ABCAM) (1:2000 dilution). The intensity of bands in western blot to analyze relative expression level of MeFDH1 was quantified by using Gel-Doc system (Gel-Doc XR system, Bio-Rad).

### Electrochemical CO_2_ reduction system and formate analysis

Electrochemical CO_2_ reductions were performed as previously reported^19^. The electrochemical reduction system consisted of a copper plate cathode (2 × 1.5 cm), a reference electrode (Ag/AgCl), and a platinum wire as anode electrode. During CO_2_ reduction reactions, the platinum wire submerged in 1 mM H_2_SO_4_ aqueous solution (initial volume: 10 mL) and generated both electrons and protons, which passed through a proton-exchange membrane (Nafion®115, 0.005-inch thickness, 30 × 30 cm, Sigma-Aldrich, USA) to the cathode section. The cathode section (initial volume: 10 mL) with 0.6 g of wet-cell as whole-cell biocatalyst, 200 mM potassium phosphate buffer (pH 6.0) and 10 mM methyl viologen (MV) worked to reduce CO_2_ to formate by utilizing electrons and protons supplied by the anode section. The cathode section solution was saturated with high purity CO_2_ gas (99.999%, purging rate: 1 mL/s) and stirred at 300 rpm at room temperature. When the Ag/AgCl electrode (MF-2079, BASi) was used as reference electrode, the electric potential (−0.75 V) of redox was constantly controlled by a potentiometer (MultiEnStat3, PalmSens, Netherlands). The concentration of formate produced by whole-cell catalysis reaction was analyzed with HPLC. HPLC analysis was performed at 30 °C and used a refractive index detector (RID) with an Aminex HPX 87-H Ion Exclusion Column (300 × 7.8 mm, Bio-Rad). The mobile phase was 5 mM H_2_SO_4_ (0.6 mL/min).

## Electronic supplementary material

supplementary information
