# Supplementary material for: Bioelectrochemical conversion of CO2 to value added product formate using engineered Methylobacterium extorquens
Source: Sci Rep. 2018 May 8;8:7211. doi: 10.1038/s41598-018-23924-z (PMC5940731; doi:10.1038/s41598-018-23924-z)
Supplement: Supplementary file 1 — supplementary information [file 41598_2018_23924_MOESM1_ESM.pdf]

Supplementary Information

Bioelectrochemical conversion of CO_2 to value added product formate using engineered *Methylobacterium extorquens*

Jungho Jang[1], Byoung Wook Jeon[1], and Yong Hwan Kim[1]*

[1]School of Energy and Chemical Engineering, Ulsan National Institute of Science and Technology(UNIST), 50 UNIST-gil, Ulsan, 44919, Republic of Korea

*Corresponding author

Yong Hwan Kim

e-mail: metalkim@unist.ac.kr

Phone: +82-52-217-3068

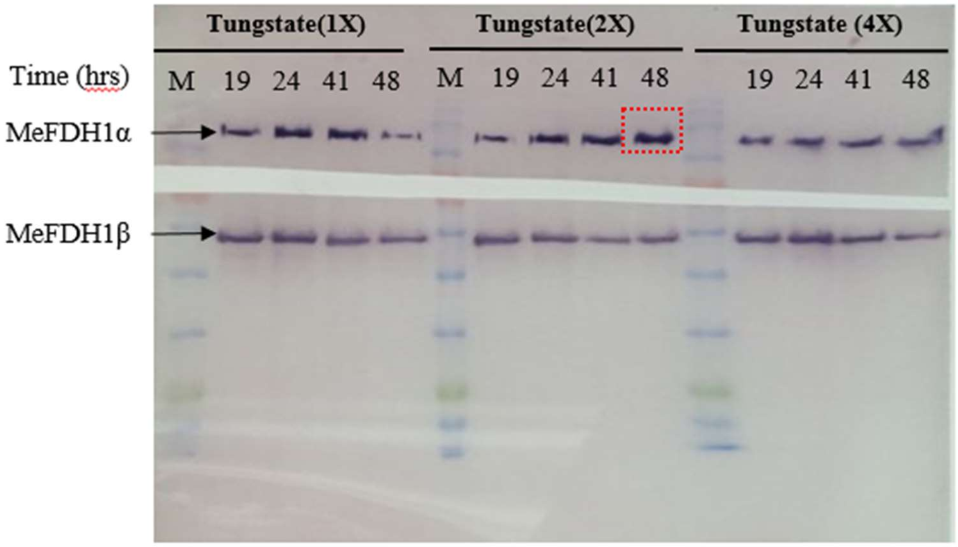

Figure S1. Western blotting of mutant (F1A-P1) crude extracts when cultured in 1x tungstate (30 μM), 2x tungstate (60 μM) and 4x tungstate (120 μM): protein marker (M), incubation times (19hr, 24hr, 41hr, 48hr).

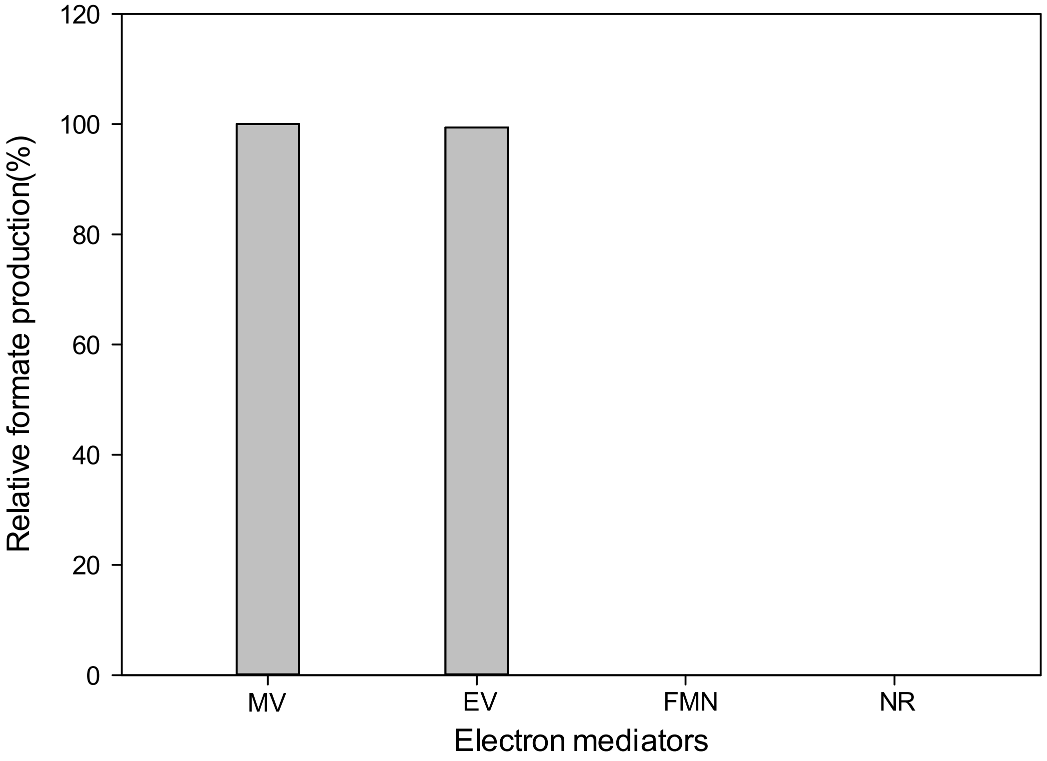

Figure S2. The relative formate production of the mutant (F1A-P1) with different type of electron mediators in electrochemical CO_2 reduction system: methyl viologen (MV), ethyl viologen (EV), flavin mononucleotide (FMN) and neural red (NR).
